# Bridging the gap between basic science and clinical practice: a role for community clinicians

**DOI:** 10.1186/1748-5908-6-34

**Published:** 2011-04-04

**Authors:** Katherine Kahn, Gery Ryan, Megan Beckett, Stephanie Taylor, Claude Berrebi, Michelle Cho, Elaine Quiter, Allen Fremont, Harold Pincus

**Affiliations:** 1 RAND Health, Santa Monica, California, USA; 2 Department of Medicine, David Geffen School of Medicine at UCLA, Los Angeles, California, USA; 3 RAND Health-University of Pittsburgh Health Institute, Pittsburgh, Pennsylvania, USA; 4 Department of Psychiatry, Columbia University, New York, New York, USA; 5 Division of Quality and Safety, New York-Presbyterian Hospital, New York, New York, USA; 6 Compass Lexecon, Oakland, California, USA; 7 UCLA School of Public Health, Los Angeles, California, USA

## Abstract

**Background:**

Translating the extraordinary scientific and technological advances occurring in medical research laboratories into care for patients in communities throughout the country has been a major challenge. One contributing factor has been the relative absence of community practitioners from the US biomedical research enterprise. Identifying and addressing the barriers that prevent their participation in research should help bridge the gap between basic research and practice to improve quality of care for all Americans.

**Methods:**

We interviewed over 200 clinicians and other healthcare stakeholders from 2004 through 2005 to develop a conceptual framework and set of strategies for engaging a stable cadre of community clinicians in a clinical research program.

**Results:**

Lack of engagement of community practitioners, lack of necessary infrastructure, and the current misalignment of financial incentives and research participation emerged as the three primary barriers to community clinician research participation. Although every effort was made to learn key motivators for engagement in clinical research from interviewees, we did not observe their behavior and self-report by clinicians does not always track with their behavior.

**Conclusions:**

A paradigm shift involving acknowledgement of the value of clinicians in the context of community research, establishment of a stable infrastructure to support a cohort of clinicians across time and research studies, and realignment of incentives to encourage participation in clinical research is required.

## Background

Translating the extraordinary scientific and technological advances from the biomedical research laboratory into actual patient care practices and other processes aimed at promoting health has been a major challenge, particularly for patients seen in community settings. In 2003, in an effort to address this challenge, the National Institutes of Health (NIH) developed the Roadmap for Medical Research, a framework of the priorities endorsed by the NIH to optimize its entire research portfolio [1]. Recommendations for addressing the challenges have included improving the public and political dialog about science [2], recruiting, training, and retaining additional clinical research scientists [3,4]; and finally, reconfiguring the scientific workforce [1,5] to bring communities and community clinicians into the mainstream of the national clinical research enterprise,[2,3,6-8], which has traditionally been dominated by clinicians and scientists at academic medical centers, federal and other research centers, and pharmaceutical companies.

Increasing participation of community clinicians in clinical research would have a number of benefits. First, the applicability and relevance of clinical research to the community practitioners who deliver most American healthcare services and to their patients would be improved by deriving data from community populations similar to those to whom evidence-based principles of care will be applied. Improving the applicability of clinical findings would motivate clinicians to increase adherence to evidence-based practices, improving survival and health-related quality of life [9-11].

Second, as experts in the delivery of clinical care in community settings, community clinicians have much to contribute. They provide care for patients across the spectrum of disease, are among the first to recognize changes in patients' needs associated with shifting demographics and burden of illness, and see patients in proportion to the prevalence of conditions in our communities. Clinicians are at the forefront of patient care associated with unexpected events such as trauma, natural disasters, and pandemic infections. They are also directly affected by policy-related matters (*e.g.*, health consequences associated with changes in pharmacy benefits or the clinical consequences of war, such as the rapid rise in the number of individuals with prosthetic limbs and post traumatic stress syndrome). Further, given their broad set of skills, the involvement of community clinicians with research could facilitate the identification, design, and implementation of research in the community on a scale that could make a difference to the American population.

Finally, participation in clinical research would benefit community clinicians in a variety of ways. Table 1 outlines these benefits, such as: contributing to the mission of medicine and improving the scientific basis for the practice of medicine; allowing clinicians to remain current with new innovations; and developing information systems to improve data-gathering associated with research. Yet, if meaningful expansion of practice-based research in community settings is to occur, it is essential to understand the reasons why only 3% of all clinicians participated in research as of 2004 [12], and to develop strategies to facilitate research in community settings. No accurate source for the total number of community investigators could be identified in the literature, but a count of clinicians included in the Federation of Practice-Based Research Networks' November 2006 Inventory of Networks revealed approximately 9,750 physicians (1 to 2% of US physicians) as members [13].

**Table 1:** Benefits to community clinicians associated with their participation in clinical research

Type of Benefit	How Clinicians Benefit
Benefits to the profession of clinical medicine and associated specialty organizations	• Contributes to the mission of medicine and improves the scientific basis for the practice of medicine.
	• Facilitates clinicians' gaining support from professional organizations and NIH.
	• Allows clinicians to contribute to the development of new knowledge; research participation provides a mechanism for this to take place.
	• Support clinicians to learn and implement what's best for their patients.

Benefits to clinicians in their role as clinicians	• Allows clinicians to remain current with new innovations.
	• Affords intellectual stimulation, an often-welcomed change from demands of clinical practice.
	• Promotes affiliation with desirable colleagues and belonging to a community with other professionals.
	• Provides a second income stream, which diversifies financial risk.
	• Generates prestige of being engaged in research and professional recognition (*e.g.*, authorship, wall certificates, or CME credits).
	• Offers free medications and/or diagnostic and therapeutic interventions to participants of some studies.

Benefits to clinicians in their role as managers of their practice settings	• With patients wanting more and payors giving less, clinicians have much to gain from evidence-based studies that delineate care known to improve outcomes.
	• Many patients seek out clinicians who are at the cutting edge of research and thus provide access to the best diagnostic and treatment options, some of which may otherwise be unavailable.
	• Participation in clinical research serves as a marketing tool for clinicians to signal their clinics as outstanding.

Benefits to clinicians in their interactions with managed care	• Clinician participation in research builds infrastructure that can facilitate engagement with managed care.
	• Information systems improve with the data gathering and transfer associated with research.

Benefits to clinicians in their efforts to improve the health of community populations	• Participation by a diverse set of clinicians and their patients is most likely to illuminate relationships between care and outcomes that apply to most patients seen in community practice.

Since 2006, NIH has funded Clinical and Translational Science Awards (CTSA) at 55 academic institutions in 28 states with a goal of 60 institutions by 2012 when the project will be fully implemented and linked [14,15]. A major goal of the CTSA program is the development of teams of investigators from a variety of research disciplines who can take scientific discoveries from the laboratory and turn them into treatments and strategies for patients in offices and communities. However, even with their introduction only a small proportion of community providers actually participate in clinical research. The goal of this research is to identify the barriers and propose solutions to challenges associated with engagement of community clinicians to facilitate current and future CTSA and other community researcher participation in medical research. In addition to the NIH endorsement, value associated with community provider participation is becoming more apparent [16,17]. As a component of the NIH roadmap, consideration was given to the development of a sustained cadre of large numbers of practicing clinicians who could participate in clinical research in the context of their community practice [1,2]. We were funded by NIH to develop a conceptual framework as a model for a system that would allow a large number of clinicians to participate in clinical research while they care for patients in their office settings. To assist NIH in the development of a conceptual framework as a model for this new type of infrastructure for translating research into practice and back [2], we conducted a classic formative evaluation [18-21].

This manuscript addresses the challenges that must be addressed to motivate community clinicians to commit to a sustained engagement in research in the settings in which they deliver clinical care. As part of the effort supported by the NIH roadmap, we conducted a study to identify the feasibility of a new national cadre of practicing clinicians who could participate in clinical research in the context of their community practices by focusing on barriers to research participation and strategies to overcome them. This paper describes the barriers reported by community clinicians and proposes potential strategies for avoiding them.

## Methods

### Overview

We used an iterative process to focus the content of interviews to best assess the perspective of clinicians and other key stakeholders regarding the feasibility of ongoing research participation by community clinicians in their own practice settings. Clinicians included physicians, dentists, and nurse practitioners. Other stakeholders were defined as individuals who led or coordinated research operations associated with clinical research or clinical practice networks, and representatives of organizations that recruit, train, or support community clinician involvement in clinical trials and/or clinical research networks. We began with an environmental scan of academic and trade journals, the internet, and public- and private sector reports of clinical and community-engaged research. The results of this review were used to develop semi-structured interview protocols that varied somewhat according to the interviewees' experience with clinical research. The protocols served as a general guide with example probes rather than as a set of specific questions to be asked of every respondent.

We interviewed key informants who could provide information about the realities of clinical research and clinical practice in terms of opportunities, costs, and liabilities based upon informants' clinical, research, and/or administrative and leadership experiences. From September 2004 through August 2005, the evaluation team conducted key informant interviews of clinicians and other stakeholders to assess the feasibility of implementing research in the context of ongoing community practices. Using a two-phase process, we initially developed a preliminary list of candidate organizations and individuals who could potentially provide information about the feasibility of adapting healthcare delivery systems and clinical practice to support clinical research in community practices. After review of candidate bios, publications, and references pertinent to their clinical, published, or administrative (leadership), for phase one interviewers, we identified a set of candidate informants to provide information about the feasibility of the program. For phase two interviews, we then conducted interviews relevant to the feasibility of a program of community-based clinical research overall and within specific urban and rural settings across all regions of the US.

### Participant interview methods

The initial approach to obtaining stakeholder input began with a focus on four key groups of stakeholders whose representatives we expected could provide unique information regarding the incentives, disincentives, and barriers to clinician enrollment of their patients in clinical research. The four key groups include:

1. Individuals (community clinicians, study leaders, and study coordinators) who already participate in clinical research networks.

2. Community clinicians in whose office clinical research could potentially take place, though they have no prior history of participating in research.

3. Professionals directing clinical research networks involving research in community practices that could serve as prototypes for research in community settings.

4. Representatives of professional societies, pharmaceutical companies, clinical research organizations, and other organizations that have recruited and trained community healthcare providers for clinical trials and clinical research networks and/or have key information regarding clinical research networks.

Our expectation was that these representatives could provide unique information regarding the incentives, disincentives, and barriers to clinicians' enrollment of their patients in clinical research. We considered each representative to be a key stakeholder whose input into and support of various facets of a clinical research program within their community would contribute to its success.

### Identification of participants and data sources

We sought not only to identify a reasonable number of informants in each major category (*e.g.*, providers) and subcategories (*e.g.*, primary care-based research networks) but also to ensure that the sample was diverse with respect to geography, informant demographics, knowledge, and experience base. We also focused on those informants who could provide data on specific costs of conducting clinical research.

We used key contacts supplemented by 'snowball' sampling in which we asked each informant to identify additional individuals we should interview from selected categories [22]. This snowballing was an iterative process in which new leads from interviews and continuing feedback from the NIH project officer continually expanded the number and types of informants identified. At the same time, our targeting of specific individuals to interview was informed by emerging themes and issues for which we believed additional interviews with representatives from a given stakeholder group would be helpful.

### Interview protocol development and use

We developed interview protocols to learn informants' views about the feasibility of various design strategies for supporting research in clinical practice in community settings. Interview protocols served as a general guide with example probes rather than as a set of specific questions that was asked of every respondent. Examples of major topics addressed in various protocols are shown in Additional File 1.

### Interview data collection process

All informants prior to being interviewed were sent descriptive materials about the NIH roadmap initiative and the proposed concept of NIH possibly launching a program to support the conduct of research within community clinical practice, the purpose of the interviews, and a consent protocol. The RAND institutional review board (IRB) reviewed these materials and procedures prior to the start of the interviews. The one-page consent protocol that had been mailed to informants in advance of the interviews was orally read verbatim to interviewees at the beginning of the interview phone call. Informants were asked to agree to participate prior to participating in the body of the interview.

One or more of the investigators on the project conducted each interview, each with an advanced academic degree associated with interview training. In all, seven team members led and/or participated in the interviews. All interviews were audiotaped and transcribed into text. Transcriptions were read and checked for accuracy by the primary interviewer.

At the conclusion of each interview, the interviewer(s) identified any key themes and issues raised during the interview. In addition, all investigators participating in interviews attended weekly debriefing meetings in which key themes and unresolved issues were discussed. This approach served not only to facilitate rapid sharing of new information and themes that were identified but also to identify issues that should be further explored in upcoming interviews.

### Analysis of interview data

All interview transcripts were entered into a text management software program (*Atlas/ti*). Two or more investigators reviewed all transcripts within two weeks of the interview to identify key themes. Each reviewer compiled an independent list of initial themes. These were then reviewed by the research team (including all interviewers) and a consensus was reached as to which themes to examine more fully. A codebook was then developed and applied to all the transcripts. In this exploratory phase, it was most important to check to ensure that the main themes were endorsed by our informants. To this end, we check with almost one-quarter of the phase one informants during a follow-up interview where we confirmed that our selected themes were indeed salient to our informants [23]. This attention to detail resulted in a key issues content change between the early and the late interviews that is specified in Table 2. The interview findings and the literature review informed the development of a model for a program to recruit and train a stable group of community clinicians for participation in research. Based on these findings, we proposed a set of tactics and strategies to address the barriers which the community clinicians identified and further refined the model.

**Table 2:** Content of interviews and types, and numbers of interviewees

**Early phase one interviews**^**a**^	**Later phase two interviews**^**b**^	**Type of interviewee**^**c**^	Number of interviews (total = 243)
Incentives and disincentives for provider participation, including organizational barriers and motivators	Best practices in community research networks, and how new provider networks might partner with these	Community clinicians (Individual primary care clinicians, dentists, nurse practitioners) and clinician organizations (health plans, large community practices) not currently participating in research	37
Strategies for provider participation and retention	Proposed provider effort as complementary to or in competition with existing clinician organizations; Liability and marketing concerns	Individual clinicians and health provider organizations already participating in clinical research	30
Ethical and professional issues	Optimal design for studies in community practices; Costs associated with conducting various types of clinical research studies in community settings	Leaders and coordinators of clinical research networks (*e.g.*, CCOPs, AMC leaders, PBRNs)	80
Advantages and limitations of different types of research networks/organizations by study and provider type and the potential role of emerging information systems	Governance, oversight, and quality control for NCRA	Representatives of private-sector organizations (*e.g.*, CROs) and stakeholders (*e.g.*, professional associations, pharmaceutical companies) with relevant experience and interest	77
Specific recommendations to NIH on design of physician recruitment and incentives	Addressing privacy, HIPAA and institutional review boards issues	Representatives of public and government entities (*e.g.*, leaders from NIH institutes and other federal agencies) with relevant experience and interest	19

^a ^A list of key issues discussed during early phases of interviews. See Appendix 1 for list of early phase interview informants.

^b ^A list of key issues discussed during later interviews, after review of transcripts of early interviews. See Appendix 2 for list of later phase interview informants.

^c ^Interviewees were selected from a listing developed by key stakeholders, authors of pertinent publications, recommendations by national organizations, and by recommendations by NIH Institute Leaders. Contact with members of this list, supplemented by snowball sampling, was used to generate the list of interviewees table.

## Results

### Interview participants

Between September 2004 and September 2005, a total of 243 informants representing affiliations from a broad collection of settings that varied with respect to practice type, size, ownership, and access to technologies such as electronic medical records and web-based research tools, were interviewed. Interview participants were diverse by advanced degree (MD 70%, PhD 10%, DDS 6%, MD PhD 4%, RN nurse practitioner 3%, Master's degree 3%, and unknown 3%). Thirty percent of participants were female. Participants came from 35 different states.

For phase one interviews, we identified 106 potential informants and attempted interviews with 97. Amongst those attempted, we completed 73 (response rate 73/97, or 75%). Of those not interviewed, six declined to participate, one was unavailable, and 17 did not respond. Additional File 2 shows the different categories of informants and the number of each type interviewed during phase one. In many cases, respondents could be placed in more than one category, but for the purposes of this report we list only their primary role.

For phase two interviews, we identified 237 potential informants and attempted interviews with 204. Amongst those attempted, we completed 170 (response rate 170/204, or 83%) including interviews with 112 active clinicians. Fifteen informants participated in more than one interview. Of those who invited to participate, 16 of 33 community providers referred to the research team by a practice-based research network (PBRN) contact specifically for interview, declined because of their busy schedules. Additionally, four other invited participants were unavailable, and 19 did not respond. Additional File 3 shows the different categories of informants and the number of each type interviewed during phase two.

Across both phase one and phase two interviews, there were many cases in which respondents could be placed in more than one participant category. However, we categorize respondents only according to their primary role. For example, in addition to the 44 phase two providers listed below, 10 more clinicians with active practices were interviewed but are categorized as PBRN leaders, rather than clinicians. Several academic medical center leaders (>8) also maintain active clinical practices.

### Interview themes documenting factors impeding clinician participation in research based in community settings

The factors identified that impede clinician participation in community-based research fall into three categories: the need for greater attention on the part of the research community to address concerns unique to community practitioners; the absence of necessary infrastructure; and the current alignment of financial incentives. Below, we introduce each of these categories with a quote from informants and then provide multiple dimensions of the factors as identified by informants. We address each of these perceived barriers along with possible strategies to overcome them in Table 3.

**Table 3:** Barriers clinicians have identified regarding participation in clinical research

**Addressing professional values**:	
Study questions	**Study questions are not pertinent to topics of interest for clinicians, their practice, or their patients**.
Study design feasibility	Study inclusion and exclusion criteria make most community practice patients ineligible.
Clinician's relationships with clinical/scientific communities	Clinicians need reassurance that research engagement does not threaten the doctor-patient relationship.
Clinician and patient distrust of research	Equitable access to research opportunities & to care reflecting research findings will help address longstanding mistrust by clinicians and patients for research endeavors.
**Developing necessary infrastructure**:	
Data quality	Assuring data quality in office settings is challenging, particularly given the lack of uniformity of study design across studies.
Design efficiency	Adequate and efficient training for successful research participation is not readily available or pertinent to clinician practice settings.
Study costs	Costs and effort associated with transient research engagement are excessive.
Research training	Local research training efforts are not rigorous enough.
Assuring privacy	Accessing IRB and HIPAA certification is burdensome and time-consuming.
Research engagement	Research participation is isolating without systematic feedback about performance, data quality, and research findings.
**Realigning financial incentives**:	
Scheduling	There is no time to do research in a busy practice.
Reimbursement	Clinical research participation will not be reimbursed adequately.
Liability	The adequacy of legal liability (insurance?) for research participation for practicing clinicians is murky.
Predictability	Unpredictable nature of research (sporadic study availability, changes in costs and reimbursement rates).
Information availability	Information is not readily available (study questions, protocols, reimbursement schedules, study-specific enrollment, data quality).

### Category one: Need for greater attention to concerns of community practitioners

'If clinicians are recruited to participate in research activities and their participation is seen as valuable, as opposed to just being a passive partner for a study, then they'll come to the table to help with the conceptualization and contribute to the science and all of the project.' Quote from a clinician involved with research in community clinical practices settings.

### Addressing community practice concerns

Clinicians as a group repeatedly expressed the belief that without acknowledgement of their potential contribution (via non-fiscal or fiscal recognition), they have little stake in clinical research and will not contribute in a sustained manner. When clinicians believe their voices are heard and responded to, they have more of a stake in clinical research and are more willing to respond to the inevitable challenges that arise.

Some of the mechanisms that were suggested by respondents to engage clinicians included: reframing research questions and study designs to increase meaning for community clinicians; attending to the complexities of the relationships between community and academics, which can become magnified in research studies; and addressing clinician and patient distrust of research.

### Study questions

Both clinicians and research leaders indicated a mechanism is needed to identify and focus on research questions that are of interest to community-based clinicians and patients, which could help close the gap between the existing research enterprise leadership and clinicians.

Physician participation in clinical research ultimately depends upon their belief that the research will benefit them and their patients.

### Study design

Community clinicians repeatedly voiced their views that they have important contributions to make about which study designs are likely to be feasible in their practice settings.

Community clinicians indicated their interests would likely be better captured when study designs generate evidence to inform the complex clinical decisions practitioners like themselves make in their practices.

Table 4 compares two categories of study designs pertinent to clinical research in community settings: explanatory and participatory (or practical) research trials. Combining explanatory and participatory trials may be an effective strategy for including community practice settings in research that aims to bridge the gap between basic science (*e.g.*, the mapping of the genetic code) and clinical applications. Placebo controlled studies, may not be a viable option in some community practice clinical situations. However, trials can be designed to compare two different treatments of the same modality (*e.g.*, comparing an investigative medication to a standard treatment for the same indication), two treatment modalities (*e.g.*, medication vs. counseling), or one modality versus both [24-26]. Trials also can be designed to inform the extraordinary challenges associated with translating the results of clinical research into clinical practice, ranging from the need to evaluate whether treatments shown to be efficacious in clinical trials continue to be effective in real-world practice, and the need to better understand the best ways to implement effective treatments across a wide variety of settings. The following sections describe strategies that are available to respond to concerns clinicians voiced regarding the evaluation of the implementation of effective strategies in community settings.

**Table 4:** Explanatory and practical clinical trials: Two options for clinical trials in community settings [7,42]

	**Explanatory clinical trials**:	**Practical clinical trials**:
**Hypothesis and design**	Hypothesis and study questions are designed to improve the understanding of the mechanism by which an intervention works	Hypothesis and study questions are designed to facilitate decision making
**Research question**	How effective is a treatment under ideal, experimental conditions?	How effective is a treatment in every-day practice? What are the risks, benefits, and costs in every-day practice?
**Defining the patient sample**	Rigorous inclusion/exclusion criteria to create a well-defined, homogenous sample of patients	Wide inclusion/exclusion criteria to reflect actual, often diverse, patient populations in clinical practices
**Practice setting**	Homogeneous	Many and diverse
**Intervention**	Well-specified, precise protocol with limited variation allowed; often involves treatment vs. placebo	Well-specified, precise protocol allowing variation in implementation from site to site to capture actual patient and care characteristics; often compares existing, clinically-relevant, feasible treatment alternatives (often head-to-head)
**Adequate sample size**	Enough to assemble a homogenous group that will enable a study of a relationship between a single intervention and a dominant outcome measure	Often requires large sample size to account for heterogeneity in sample and long-term nature of studies
**Outcome**	Well-defined; often a specific biological effect of an intervention	Often defined broadly in relation to patient's function or quality of life so effect sizes on personal and population health can be calculated

### Concerns pertinent to the clinician's relationships with the clinical and scientific communities

Community clinicians expressed concerns that research participation might result in shifts in patient management from their own to academic practice settings.

To alleviate such concerns, study designs can specify that patients identified for research participation will maintain their relationships with their own clinicians for the bulk of their care.

### Clinician and patient distrust of research

Despite the extraordinary advances in clinical care and outcomes that have emanated from research, longstanding distrust has also accumulated. Years after the Public Health Service (PHS) Syphilis Study in Tuskegee, Alabama, practicing clinicians expressed concerns that many patients remain mistrustful of research, fearing that risks are not fully disclosed, benefits may be exaggerated, and health information may be mishandled [27-29]. Accordingly, many clinicians are reluctant to discuss potential research opportunities with their patients.

Expansion of research into community settings will need to build on recent efforts to improve trust and safety for patients engaged in community research efforts [30]. Facilitating equitable and diverse involvement of clinicians and patients in clinical research can help achieve these goals as described by the Council of Public Representatives (COPR)[31].

### Category two: Absence of infrastructure

'A lot of the research can't necessarily be turned over entirely to the practices and the practitioners because they just don't have the manpower or the infrastructure. When we're able to send people out into the practices it's actually very helpful. What an expanded, national network can do is identify a study coordinator for each participating practice in their network. That person may or may not be one of the physicians. More often it's a staff, possibly a nurse or a clerk. They're responsible for the day-to-day operations of the project. When they are long-term, that provides a critical infrastructure ingredient for success.' Quote from an experienced research administrator who has led research in a variety of community settings.

### Necessary infrastructure

Clinicians expressed concern regarding the lack of a permanent infrastructure to assist with identifying and choosing appropriate research opportunities, acquiring necessary data collection and other research skills and equipment, working with institutional review boards, and handling other challenges they would face if they agreed to participate in research.

Organizational supporting structures will need to become flexible enough to allow clinicians a voice in essential practice and other local decisions, while remaining durable enough to assure continued high-quality research endeavors. Resources for such an infrastructure will need to be allocated in advance, and initial costs will be high. Both central (*e.g.*, NIH, national specialty societies) and local organizations could provide support, but in exchange, a cost-effective, stable research infrastructure will need to be established [7,8,26,32,33].

### Flow of information to clinicians

Clinicians cited not only a lack of knowledge about pending research opportunities but also a lack of information needed to make an informed decision about participation (both the appropriateness of their practices and their ability to accept such a commitment). To alleviate these concerns, the clinical research enterprise could provide a reliable and sustained flow of information about research opportunities and eligibility requirements. NIH has already launched their landmark effort to provide information on NIH-funded clinical trials to patients and clinicians [34].

When clinicians have access to study questions and protocols before agreeing to participate, they can choose protocols they can successfully implement. A trial registry modeled upon the NIH Clinical Trials registry is one mechanism for providing potential clinician participants with regular updates, including study-specific enrollment and other information that would help them gauge the quantitative and qualitative value of their participation in studies. A clinical trial registry would also provide a venue for sharing trial data among participants of ongoing trials, potentially improving the quality of data reporting and ultimately the quality of research. Improving research quality, in addition to ensuring human protection and safety, may renew public confidence and trust in the clinical research enterprise. Quality control of community practice-based research will have to be rigorous throughout the research process. Inclusion of clinicians in a quality assurance system will make use of their knowledge of their own patients and their familiarity with their practice's operations, thus enabling design of a reliable and valid data collection and monitoring process that will work within their practice, while also demonstrating transparency to outside stakeholders. Uniform standards of training, credentialing, and quality oversight will be key.

### Easing the burdens of IRBs and the Health Insurance Portability and Accountability Act (HIPAA)

Currently, all studies must undergo IRB approval, and high-risk studies receive ongoing review by a data safety monitoring board. Interviewees expressed many concerns about the challenges of navigating IRB requirements. To address such concerns, several leadership groups have called for the formation of a more standardized and centralized IRB, which has now occurred at the National Cancer Institute [33,35].

Translational research will require more clinical training and mentoring on the research process and data integrity. Additionally, we will need to implement an extensive and rigorous system of accountability to assure proper human protection and safety. A centralized web-based process to provide HIPAA and IRB training and certification and a web-based registry of certified clinicians has already become standard in many settings. Ensuring accountability at the local level will involve the research sponsor, the principal investigator, data centers, laboratories, pharmacies, and clinicians as well as an organization with responsibility to assure real time accountability. Providing clinicians with standardized training in general and study-specific principles of research with a particular focus on reporting adverse outcomes will enhance the function of this process.

### Category three: Current (mal)alignment of financial structures

'I think it does probably come down to being able to at least offset their time that's involved. And as you pointed out before, there may be some additional personnel costs that go above and beyond what the typical office personnel can handle in terms of patient education, monitoring forms, all the rest. So that's got to be part of the cost of doing credible clinical research.' Quote from an experienced clinician who has led research in community settings.

### Realigning financial incentives

Voiced repeatedly in our interviews was the complaint that in the competitive and productivity-driven environment in which clinicians practice medicine today, research participation that disrupts patient flow, decreases staff efficiency, or otherwise threatens the economic viability of a practice, discourages further interest in participation.

Although non-financial incentives such as prestige, personal satisfaction, and improved patient care motivate clinicians to participate in research, these incentives cannot entirely substitute for financial compensation for services [36,37]. When clinicians are only minimally involved with research, removal of the strongest disincentives (*e.g.*, burdensome inclusion/exclusion criteria and inefficient data collection strategies) is likely to be appreciated. However, as the level of clinician effort and participation increases and clinicians become more familiar with clinical research, interviewees stated that monetary compensation becomes a stronger incentive than clinical research support measures.

### Fair market compensation of clinicians' time

Currently, NIH-funded research relies heavily on clinician volunteerism and non-financial incentives [36]. Developing and adopting a transparent and equitable system for compensation of clinicians' time and efforts in clinical research participation could result in a more stable cohort of research clinicians. Compensation could be guided by a principle of replacement value, compensating clinicians as if they were engaged in clinical care instead of research (for the equivalent effort and time) [38].

### Cost reimbursement for clinical activities

Clinicians considering research opportunities cited uncertainties regarding costs that they or insurers would be asked to bear. Setting and revealing reimbursement schedules for most common research tasks could greatly reduce financial uncertainty and help clinicians make informed participation decisions. A relative value scale (RVS)-like system with associated clinical payment structure [38] that accounted for cost variation by geographic region, specialty, and clinical and prior research experience would allow sponsors, principal investigators, and research-associated organizations (such as PBRNs or academic research organizations) to better understand the financial costs and benefits of a research protocol before making a commitment. Further, protocols, budgets, and expected payments that estimate the actual cost of the activity would allow community clinicians to anticipate whether research participation would fit the patient care flow in their practice settings.

### Improving predictability of research activities

A related concern clinicians cited was the lack of predictability of research activities both within one study and from one study to the next, resulting in problems with budgeting, management of time, space, staffing, and monitoring. If a practice can anticipate long-term research participation, it is more likely to adjust workflow to accommodate research than if it anticipated participating in only a single or occasional study. Table 5 offers strategies to improve the predictability of clinical research.

**Table 5:** Strategies to improve the predictability of research

Strategies	Requirements
Make research-associated tasks explicit to clinicians prior to their agreeing to participate in a study	• Training requirements
	• Mechanism for patient screening to determine study eligibility
	• Inclusion and exclusion criteria
	• Number of subjects stratified by clinical, demographic, & geographic categories
	• Expected patient visits and follow-up requirements
	• Data collection and transfer strategies
	• Adverse outcome protocols
	• Quality assurance requirements
	• Dissemination
Establish *a priori *the task-specific reimbursement rates for studies	• Work with researchers & clinicians to establish a list of key research tasks
	• Develop a taxonomy for assigning payment to these tasks
	• Develop payment rates based upon specialty, experience, & region
	• Assure clinicians are clear about study-specific-protocol services
	• Implement serial evaluations to test the payment rates

### Liability

Clinicians emphasized that assuring adequate and appropriate liability coverage is mandatory before they can actively participate in community research [39]. Such assurances require coordination with clinical malpractice carriers and the coverage strategies used by research sponsors. A plan to address clinician liability coverage is most likely to achieve success if research sponsors and insurers collaborate with the private sector to address the needs of community clinicians. The distribution of costs and risks among research sponsors, clinicians, and patients, will need to ensure that research-related costs will not be born by either the physician or the patient [24].

## Discussion

Translational research - *i.e.*, research aimed at optimizing the ways in which biomedical and clinical research are linked with clinical practice and diffusion to community settings - provides an unprecedented opportunity for practicing clinicians to improve the health of Americans. Engagement of clinicians in the research enterprise will allow the extraordinary results of the basic, explanatory research conducted in recent decades to be translated into practical applications for responding to the challenges associated with major public health risks, different healthcare delivery organizations, and different types of clinicians. Clinician involvement addresses the process of applying discoveries generated during research in the laboratory, and in preclinical studies, to the development of trials and studies in human subjects, as well as research aimed at enhancing the adoption of best practices in the community.

Engaging practicing community clinicians in both aspects of translation will increase opportunities for recent medical school graduates and may encourage many clinicians who currently do not value or track developments in evidence-based medicine to begin doing so. Increased participation will lead to greater generalizability of research results, which in turn will make research more relevant to all cohorts of practitioners and build support for the research enterprise. Clinician involvement has implications for the selection of research questions, and for the conduct of effectiveness and implementation studies across diverse communities. Clinicians working with their patients can facilitate meaningful quality assurance practices related to patient inclusion and exclusion, to data gathering, and to a nuanced awareness of the fidelity of interventions occurring within settings familiar to them.

Although we made substantial efforts to interview clinician and other stakeholders with diverse clinical and research experiences as well as with varying geographic and socio-demographics characteristics, the selection of the starting point for our initial key informants for the snowball analysis could have led to the omission of significant viewpoints. However, our iterative targeting of specific individuals to interview based upon emerging themes and issues for which we felt additional interviews with representatives from a given stakeholder group would be helpful, likely mitigated any such effect.

Implementation of a variety of strategies involving both research and clinical care systems can tip the balance so that clinicians begin to perceive that the benefits from clinical research participation outweigh the barriers. Strategies such as the RE-AIM (reach, effectiveness, adoption, implementation, maintenance) evaluation framework have developed to support the assessment of interventions in terms of the translatability and public health impact of health promotion [40-42]. However, in addition to new evaluation strategies, a paradigm shift changing clinician's perceptions and involving multiple key stakeholders in both the national clinical research enterprise and clinical medicine is needed to tip the balance toward community practitioner participation in clinical research, helping to bridge the gap between basic and applied research.

## Competing interests

The authors declare that they have no competing interests.

## Authors' contributions

KK, MB, and GR designed the study and drafted the manuscript. EQ, CB, ST, MC, and HP guided study design and read and revised the manuscript. All authors have read and approved the final manuscript.

## Supplementary Material

Additional file 1 Appendix 1: Major topics addressed in interview protocols for Phases I and II. Click here for file

Additional file 2 Appendix 2: Completed phase I interviews by informant type (n = 73). Click here for file

Additional file 3 Appendix 3: Completed Phase II interviews by informant type (n = 170). Click here for file
