# Supplementary material for: Bridging the gap between basic science and clinical practice: a role for community clinicians
Source: Implement Sci. 2011 Apr 4;6:34. doi: 10.1186/1748-5908-6-34 (PMC3087703; doi:10.1186/1748-5908-6-34)
Supplement: Additional file 1 — Appendix 1: Major topics addressed in interview protocols for Phases I and II. [file 1748-5908-6-34-S1.DOC]

**Appendix 1: Major Topics Addressed in Interview Protocols for Tasks 1 and 2**

| **Phase 1** |
| --- |
| Methods of clinician recruitment |
| Incentives and disincentives for clinician participation |
| Motivations for clinician participation |
| Options for clinician participation |
| Methods for clinician retention |
| Organizational barriers to clinician participation |
| Strategies for clinician retention |
| Advantages and limitations of different types of research networks/organizations |
| Potential role of emerging information systems |
| Addressing privacy, HIPAA* and institutional review board issues |
| Specific recommendations to NIH on practice design to support research within community practices |
| **Phase 2** |
| Testing reactions to proposed research models within community practices |
| Issues related to partnering |
| Infrastructure organizations to complement clinician organizations to support clinical research within practices |
| Optimal configuration for different types of research studies |
| Governance, oversight, and quality control for research conducted within clinical practice |
| Ethical and professional issues |
| Political and liability issues |
| Costs associated with conducting various types of clinical research studies |
| * HIPAA: Health Insurance Portability and Accountability Act |
