# Supplementary material for: Bridging the gap between basic science and clinical practice: a role for community clinicians
Source: Implement Sci. 2011 Apr 4;6:34. doi: 10.1186/1748-5908-6-34 (PMC3087703; doi:10.1186/1748-5908-6-34)
Supplement: Additional file 2 — Appendix 2: Completed phase I interviews by informant type (n = 73). [file 1748-5908-6-34-S2.DOC]

**Appendix 2: Completed Task 1 Interviews by Informant Type (n=73)**

| **I. PROFESSIONALS DIRECTING CLINICAL RESEARCH AND/OR PARTICIPATING IN CLINICAL RESEARCH NETWORKS INVOLVING CLINICAL PRACTICES** |
| --- |
| Clinician Researchers: Primary care research networks (15): Includes physicians, dentists and nurse practitioners leading or serving as active members of a primary care research network. |
| Clinician Researchers: Specialty care research networks (4): Includes physicians, dentists, and nurse practitioners leading or serving as active members of a specialty research network. |
| **II. INDIVIDUAL STUDY LEADERS OR COORDINATORS EXPERIENCED WITH OPERATIONS ASSOCIATED WITH CLINICAL RESEARCH NETWORKS INVOLVING CLINICAL PRACTICES** |
| Research Administrator, Manager, or Coordinator (3): Includes individuals who serve as research coordinators or managers at the site or network level |
| Research Organization, Private (4): Includes individuals who are leaders in private clinical research organizations (CROs) |
| **III. PRACTICING COMMUNITY CLINICIANS NOT PARTICIPATING IN CLINICAL RESEARCH, BUT POTENTIALLY COULD CONDUCT RESEARCH WITHIN THEIR CLINICAL PRACTICES** |
| Physician in practice (10): Includes physicians whose primary focus is the provision of medical care within the ambulatory setting. |
| Dentist in practice (4): Includes dentists whose primary focus is the provision of dental care within the ambulatory setting. |
| **IV. REPRESENTATIVES OF ORGANIZATIONS THAT RECRUIT, TRAIN, OR SUPPORT COMMUNITY CLINICIAN INVOLVEMENT IN CLINICAL TRIALS AND/OR CLINICAL RESEARCH NETWORKS** |
| Affiliated with an Academic Medical Center (13): Includes physicians, dentists, and nurse practitioners whose primary affiliation is with an academic institution. |
| Affiliated with a Professional Organization (9): Includes individuals whose primary affiliation is with a primary care or specialty organization. |
| Affiliated with a Healthcare Delivery Organization (3): Includes individuals whose primary affiliation is with a health care delivery institution, such as a clinic or community hospital. |
| Affiliated with Health plans (serving as insurers and providers) (2): Individuals whose primary affiliation is with organizations that provide both a healthcare insurance plan and a healthcare delivery system |
| Affiliated with Health plans (serving as insurers only) (2): Individuals whose primary affiliation is with a healthcare insurer |
| Federal Agencies (3): Individuals who are National Institutes of Health (NIH) or Agency for Health Research and Quality (AHRQ) officials, including NIH Roadmap National Clinical Research Associates Subcommittee member |
| Pharma (1): Individuals who are leaders in the pharmaceutical industry |
