# Supplementary material for: Bridging the gap between basic science and clinical practice: a role for community clinicians
Source: Implement Sci. 2011 Apr 4;6:34. doi: 10.1186/1748-5908-6-34 (PMC3087703; doi:10.1186/1748-5908-6-34)
Supplement: Additional file 3 — Appendix 3: Completed Phase II interviews by informant type (n = 170). [file 1748-5908-6-34-S3.DOC]

**Appendix 3. Completed Phase 2 Interviews by Informant Type (n=170)**

| **I. PROFESSIONALS DIRECTING CLINICAL RESEARCH AND/OR PARTCIPATING IN CLINICAL RESEARCH NETWORKS INVOLVING CLINCIAL PRACTICES** |
| --- |
| *Clinician Researchers: Primary care research networks (26): Includes physicians, dentists and nurse practitioners leading or serving as active members of a primary care research network.* |
| Physicians (14) |
| Dentist (7) |
| Nurse practitioner (5) |
| *Clinician Researcher: Specialty care research networks (8): Includes physicians, dentists, and nurse practitioners leading or serving as active members of a specialty research network.* |
| Physician from a multispecialty medical group (3) |
| Physicians with academic medical practice (5) |
| **II. INDIVIDUAL STUDY LEADERS OR COORDINATORS EXPERIENCED WITH OPERATIONS ASSOCATIED WITH CLINICAL RESEARCH NETWORKS INVOLVING CLINICAL PRACTICE** |
| *Research Administrator, Manager, or Coordinator (11): Includes individuals who serve as research coordinators or managers at the site of network level* |
| Research coordinators (11) |
| *Research Organization, Private (18): Includes individuals who are leaders in private clinical research organizations (CROs)* |
| Clinical trial network leader (10) |
| Academic research organization leader (5) |
| Clinical research organization leader (2) |
| Dedicated research network leader (1) |
| **III. PRACTICING COMMUNITY CLINICIANS NOT PARTICIPATING IN CLINICAL RESEARCH, BUT POTENTALLY COULD CONDUCT RESEARCH WITHIN THEIR CLINICAL PRACTICES** |
| *Physician in practice (8): Includes physicians whose primary focus is the provision of medical care within the ambulatory setting.* |
| Physicians associated with small private practice office (8) |
| *Dentists in practice (7): Includes dentists whose primary focus is the provision of medical care within the ambulatory setting.* |
| Dentists in practice (7) |
| *Nurse practitioner in practice (2): Includes nurse practitioners whose primary focus is the provision of medical care within the ambulatory setting.* |
| Nurse practitioner (2) |
| **IV. REPRESENTATIVES OF ORGANIZATIONS THAT RECRUIT, TRAIN, OR SUPPORT COMMUNITY CLNIICAN INVOLMENT IN CLINICAL TRIAL AND/OR CLNICAL RESEARCH NETWORKS** |
| *A. Affiliated with an Academic Medical Center (36): Includes physicians, dentists, and nurse practitioners whose primary affiliation is with an academic institution* |
| Physician (29) |
| Dentist (4) |
| Nurse practitioner (3) |
| *B. Affiliated with a Professional Organization (10): Includes individuals whose primary affiliation is with a primary care or specialty organization.* |
| Primary care based practice research network (10) |
| *C. Affiliated with a Healthcare Delivery Organization (3): Includes individuals whose primary affiliation is with a health care delivery institution, such as a clinic or community hospital.* |
| Healthcare delivery, other (3) |
| *D. Affiliated with Health plans (serving as insurers and providers) (9): Individuals whose primary affiliation is with organizations that provide both a healthcare insurance plan and a healthcare delivery system (e.g., Kaiser, Fallon Clinic)* |
| Multispecialty health plan leader (4) |
| Multispecialty leader (3) |
| Professional and Healthcare Association Leaders (2) |
| *E.. Affiliated with Health plans (serving as insurers only) (5): Individuals whose primary affiliation is with a healthcare insurer (e.g., Wellpoint, United Healthcare)* |
| Health plan leader (5) |
| *F. Federal Agencies* *(17):**Individuals who are National Institutes of Health (NIH) or Agency for Health Research and Quality (AHRQ) officials, including NIH Roadmap National Clinical Research Associates Subcommittee member* |
| NIH subcommittee members (9)  NIH officials in other capacities (8) |
| *G. Pharma (10):* *Individuals who are leaders in the pharmaceutical industry* |
| Pharmaceutical (5)  Other business (5) |
